# Impacts of short-term tillage and crop residue incorporation managements on soil microbial community in a double-cropping rice field

**DOI:** 10.1038/s41598-022-06219-2

**Published:** 2022-02-08

**Authors:** Haiming Tang, Chao Li, Kaikai Cheng, Li Wen, Lihong Shi, Weiyan Li, Xiaoping Xiao

**Affiliations:** Hunan Soil and Fertilizer Institute, Yuanda Road, Furong district, Changsha, 410125 China

**Keywords:** Microbiology, Environmental sciences

## Abstract

Soil microbial community were usually reconsidered as a sensitive indicator in soil quality and soil environment change of paddy field. However, the effects of different tillage and crop residue incorporation managements on soil bacterial community under the double-cropping rice cropping system were still need to further investigated. Therefore, the impacts of different tillage and crop residue incorporation managements on soil bacterial community under the double-cropping rice cropping system in southern of China were studied by using phospholipid fatty acids (PLFAs) profile method in the present paper. The experiment included four different tillage treatments: rotary tillage without crop residue input as a control (RTO), no-tillage with crop residue retention (NT), rotary tillage with crop residue incorporation (RT), and conventional tillage with crop residue incorporation (CT). Compared with RTO treatment, grain yield of rice with NT, RT and CT treatments increased by 1.21%, 3.13% and 6.40%, respectively. This results showed that soil aC15:0, C16:0, iC17:0, C19:0c9, 10 fatty acids with CT and RT treatments were higher than that of RTO treatment, while soil C16:1ω6c and C18:1ω9t fatty acids with NT treatment were higher than that of RTO treatment, respectively. Soil G^+^ and G^−^ bacteria PLFAs contents with CT treatment were higher than that of NT, RT and RTO treatments, while the value of soil G^+^/G^−^ bacteria PLFAs with NT treatment were higher than that of CT, RT and RTO treatments. This results indicated that Richness and McIntosh indices with CT treatment were significantly higher than that of RTO treatment. Principal component analysis (PCA) results showed that the first and second principal components (PC1 and PC2) were explained 93.2% of total variance with all tillage treatments. Except C12:0, C14:0 2OH and C18:2ω6, all unsaturated and cyclopropyl PLFAs contents were belong to PC1. PC1 and PC2 were explained 88.4% of total variance with all tillage treatments. There had significantly positive correlation between soil Richness, Shannon indices and soil PLFAs, G^+^ bacteria, G^−^ bacteria, fungi contents. As a result, it were benefit practices for increasing soil bacterial community structure in the double-cropping rice field of southern China by combined application of rotary, conventional tillage with crop residue incorporation managements.

## Introduction

Soil microorganism plays an important role in maintaining or increasing soil quality and fertility, which were close related to nutrient cycling and sustainability of soil productivity^1^. Therefore, soil microbial community were usually regarded as a sensitive indicator for evaluating the varied of soil quality^2^. The soil microbe were main included soil bacteria, fungi, archaea, viruses, protozoa and so on. Furthermore, the phospholipid fatty acids (PLFAs) content would consider as biomarker for evaluated soil microbial community structure by investigate soil microbial biomass and group, such as bacteria and fungi^3^. Therefore, the PLFAs content were usually regarded an important indicator for reflecting change of soil microbial community for that they were vital composition of soil microorganism^4^.

In recent years, many studies results demonstrated that soil microbial community and diversity were close related to different field management, such as cropping system, tillage, crop residue, fertilizer regime and so on^5–7^. The diversity and community structure of soil microorganism were main affected by combined application of tillage with crop residue incorporation practice through changing soil microbe habitat characteristic, such as soil porosity, soil moisture and substrate. Some studies suggested that soil microbial community and diversity^5,8^, population^9^ with no-tillage (NT) practice were higher than those with conventional tillage (CT) practice. Helgason et al. (2009)^9^ results found that individual PLFAs content at surface soil (0–5 cm) with NT treatment were increased from 7 to 86%, compared with CT treatment. Jia et al. (2015)^10^ results showed that total soil bacterial and fungal phospholipid fatty acids (PLFAs) content at 0–5 cm layer with NT and ridge tillage treatments were 1.6 and 2.6 times higher than that of moldboard plow treatment, respectively. Wang et al. (2017)^11^ results indicated that soil microbial diversity by combined application of NT with organic practice were increased, compared with CT treatment. Li et al. (2020)^7^ results suggested that fungal PLFAs content and Shannon index of soil bacterial community with NT treatment were increased by 17% and 6%, compared with CT treatment, respectively. But there had some opposite conclusion, such as Gottshall et al. [2017)^12^ reported that soil fungal population were increased under application of conservation tillage management condition. Guo et al. (2016)^6^ results demonstrated that value of gram-positive bacteria/gram-negative bacteria ratio (G^+^/G^−^) were decreased with NT and straw returning treatment, compared with conventional intensive tillage and straw returning treatment. Therefore, soil microbial population and diversity in paddy field were changed under combined application of tillage with crop residue retention condition^11,12^.

Rice (*Oryza sativa* L.) is the major crop in tropical and subtropical monsoon climate region of Asia^13,14^. Chinese milk vetch (*Astragalus sinicus* L.) and early rice and late rice (double-cropping rice) is the major cropping system in Yangtze River Plain of China. But soil quality and soil biological fertility in double-cropping rice field were decreased under higher intensity cropping system condition. Recently, the practice of Chinese milk vetch and rice straw as organic fertilizer returning to paddy field were accepted by farmer in this region, rotary tillage and no-tillage with crop residue retention management were also accepted by farmer in this region, for that these practices were benefit for increasing soil quality, soil fertility and yield of rice^15^. However, it is still not known how about the impacts of different tillage and crop residue incorporation practice on soil microbial community and diversity in the double-cropping rice system of southern China. We hypothesized that soil bacterial community composition and diversity in the double-cropping rice field were modified by combined application of tillage with crop incorporation practice.

Therefore, to better understand the impacts of combined application of tillage with crop residue incorporation practice on soil microbial community in paddy field, the aims of this study were: (1) to investigate the effects of 6-years of continuous combined application of conventional tillage, rotary tillage and no-tillage with crop residue incorporation, and rotary tillage without crop residue incorporation on soil microbial community, (2) to analysis the correlation between soil microbial diversity and soil microbial characteristic, and (3) to choose an suitable tillage practice for paddy field in a Chinese milk vetch and double-cropping rice system.

## Materials and methods

### Sites and cropping system

The field experiment begun in November 2015. It were located in Ningxiang County (28°07′ N, 112°18′ E) of Hunan Province, China. The cropping system of this field experiment were included Chinese milk vetch (*Astragalus sinicus* L.), early rice and late rice (*Oryza sativa* L.). The more detail information about cropping system, climate condition (annual mean evapotranspiration and precipitation, monthly mean temperature) of field experiment region, soil texture and soil type, soil physicochemical characteristics at 0–20 cm soil layer before this field experiment were described as by Tang et al. (2019)^16^.

### Experimental design

The field experiment included four tillage treatments: rotary tillage with crop residue removed as a control (RTO), conventional tillage with crop residue incorporation (CT), rotary tillage with crop residue incorporation (RT), and no-tillage with crop residue retention (NT). The area of the each plot were 56.0 m^2^ (7 m × 8 m), and each tillage treatment were laid out by random complete block design with three times in paddy field. The more detail information about cropping system, tillage practice, amount of application of Chinese milk vetch and rice residue, inorganic fertilizer, varieties of rice, date of transplant rice seedling and harvest of rice, and other more detail information about paddy field practice were described as by Tang et al. (2019)^16^. Haiming Tang undertook the formal identification of the plant material used in our study. There were not a voucher specimen of this material has been deposited in a publicly available herbarium. It were ensure that our have permission to collect Chinese milk vetch, and were obtained permissions or licenses.

### Soil sampling

Soil sample at 0–20 cm layer were collected in October 2020, at the maturity stage of late rice. Soil sample were collected from paddy field in each plot by random selected from six cores. After removing rice root, small stone and visible organic material by hand, then the soil sample were divided into two parts. One part of the soil sample were passed through 2 mm mesh sieve and then store at 4 °C condition for investigate of soil chemical characteristics (e.g., soil total carbon (C), particulate organic C (POC), dissolved organic C (DOC), total nitrogen (N), available N, total phosphorus (P), available P, total potassium (K), and available K) within 2 weeks, and another part of soil sample were immediately placed in ice box and transported to the laboratory, and then stored at -80 °C condition for carry out phospholipid extraction. The soil samples were stored in the laboratory of Hunan Soil and Fertilizer Institute. Experimental research and field studies were comply with related institutional, national, and international guidelines and legislation.

### Soil analysis

#### Soil chemical characteristics and grain yield of rice

The chemical characteristics of soil sample were investigated in the laboratory. Soil total C, total N, available N, total P, available P, total K and available K contents of soil sample were measured according to the method described as by Olsen and Sommers (1982)^17^. Soil POC content of soil sample were investigated according to the method described as by Cambardella and Elliott (1992)^18^. The related detail information about investigate method for soil DOC content were described as by Jones and Willett (2006)^19^. At maturity stage of late rice, grain yield of rice were investigated from each plot and were expressed as rough (unhulled) rice at 14% moisture content.

#### Phospholipid fatty acids analysis

The more detail information about soil lipid extraction and phospholipid fatty acids (PLFAs) analysis were conducted on according to the method described as by Bossio et al. (1998)^20^. Soil fatty acid methyl esters (FAMEs) content were investigated according to the method described as by Yao et al. (2000)^21^. Soil PLFAs content were calculated following the equation, and were expressed as ng g^−1^ dry soil^21^:

$$ {\text{C}}_{{\text{x}}} \left( {{\text{ng}}/{\text{g soil}}} \right)\, = \,{\text{A}}_{{\text{x}}} \, \times \,{\text{c}}_{{\text{i}}} [{\text{ng}}]\, \times \,{\text{f}}\, \times \,{1}000/\{ {\text{A}}_{{\text{i}}} \, \times \,{\text{W}}\left[ {\text{g}} \right]\, \times \,{\text{M}}[{\text{ng}}/{\text{g}}]\} $$

where C_x_ were the PLFAs content of soil sample; A_x_ were the peak area of PLFAs; A_i_ were the peak area of internal standard (19:0 nonadecanoic acid); c_i_ were the absolute amount of internal standard in the vial (ng); f were response factor of different PLFAs (peak area to content ratio compared with internal standard; if not known, then 1); W were the weight of soil sample (g); and M were molecular weight of fatty acid (ng g^−1^).

#### Phospholipid fatty acids community structure analysis

Soil microbial community with different tillage treatments were analysis base on species and total PLFAs content of soil sample. In the present study, soil PLFAs content mainly included following specific, such as 16:0, 18:1ω9c, 10Me17:0 directive of bacteria^22^ and fungi^23^, 10Me16:0 and 16:1ω5c directive of sulfate reduction actinomycetes and bacteria^23^, respectively. Soil PLFAs content, Richness, Shannon and McIntosh indices were used to estimate the diversity of PLFAs composition. Richness, Shannon and McIntosh indices of soil sample were calculated according to the method described as by Schutter and Dick (2000)^24^.

### Statistical analysis

All statistical analyses and correlation analysis between soil microbial characteristic and soil microbial diversity were calculated in this manuscript by using SAS 9.3 software statistical package^25^. The data of every investigate items with each tillage treatment means were compared by using one-way analysis of variance (Anova) following standard procedures at the 5% probability level. The data of phospholipid fatty acids were measured by using principal components analysis (PCA) to expound major variation and covariation for individual PLFAs by using varimax rotation, which were conducted on log_10_ conversion mole percentage of individual PLFAs. Then the PCA score were measured by analysis of variance (Anova) with correlation matrix by using the SAS 9.3 software statistical package.

## Results

### Soil chemical characteristic and grain yield of rice

The soil chemical characteristic in paddy field were significant changed under CT, RT, NT and RTO treatments condition. Compared with RTO treatment, SOC content with CT and RT treatments were significant (*p* < 0.05) increased, soil available P content with NT and CT treatments were also significant (*p* < 0.05) increased, soil total N and available N contents with RT treatment were also significant (*p* < 0.05) increased, respectively. The results indicated that there had no significant (*p* > 0.05) difference in soil total K, total P and available K contents between NT, RT, CT and RTO treatments (Table 1).

**Table 1:** Effects of different tillage management on soil chemical characteristic in a double-cropping rice field.

Items	Total C(g kg^−1^)	Total N(g kg^−1^)	Total P(g kg^−1^)	Total K(g kg^−1^)	Available N(mg kg^−1^)	Available P(mg kg^−1^)	Available K(mg kg^−1^)
CT	22.81 ± 0.65a	2.18 ± 0.05ab	0.86 ± 0.02a	16.57 ± 0.04a	196.54 ± 5.61ab	14.67 ± 0.42a	95.65 ± 2.76a
RT	22.45 ± 0.62a	2.21 ± 0.06a	0.84 ± 0.02a	16.46 ± 0.04a	198.78 ± 5.67a	13.82 ± 0.39ab	92.82 ± 2.67a
NT	21.06 ± 0.57ab	2.14 ± 0.06ab	0.87 ± 0.02a	16.42 ± 0.04a	194.46 ± 5.58ab	14.73 ± 0.43a	93.47 ± 2.62a
RTO	20.28 ± 0.51 b	2.07 ± 0.04b	0.83 ± 0.02a	16.35 ± 0.04a	193.24 ± 5.46b	13.76 ± 0.36b	83.52 ± 2.41a

Values were presented as means ± SE.

Different lowercase letters in the same column were indicated significantly difference at *p* < 0.05 level.

The same as below.

*CT* conventional tillage with crop residue incorporation, *RT* rotary tillage with crop residue incorporation, *NT* no-tillage with crop residue retention, *RTO* rotary tillage with crop residue removed as a control.

**Figure 1:**
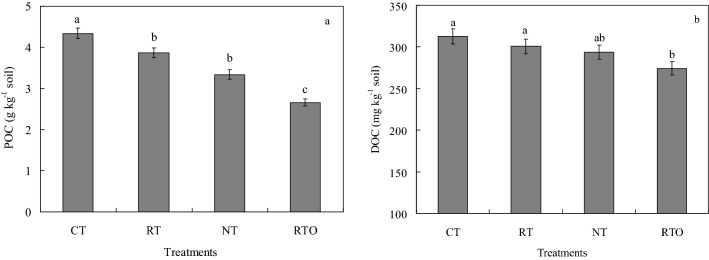
Effects of different tillage management on soil particulate organic carbon **(a)** and dissolved organic carbon **(b)** contents in a double-cropping rice field. *CT* conventional tillage with crop residue incorporation; *RT* rotary tillage with crop residue incorporation; *NT* no-tillage with crop residue retention; *RTO* rotary tillage with crop residue removed as a control. Error bars represent standard error of mean. Different smaller letters were indicated significant difference at *p* < 0.05 level.

The results indicated that range of soil POC content with all tillage treatments were varied from 2.66 to 4.34 g kg^−1^. POC content with CT, RT and NT treatments were significantly higher (*p* < 0.05) than that of RTO treatment. Compared with RTO treatment, soil POC content with CT, RT and NT treatments increased by 63.16%, 45.49% and 25.56%, respectively (Fig. 1a). The results showed that range of soil DOC content with all tillage treatments were varied from 274.39 to 312.45 mg kg^−1^. Compared with RTO treatment, soil DOC content with CT and RT treatments were significantly (*p* < 0.05) increased, soil DOC content with CT and RT treatments increased by 13.87% and 9.56%, respectively (Fig. 1b).

**Figure 2:**
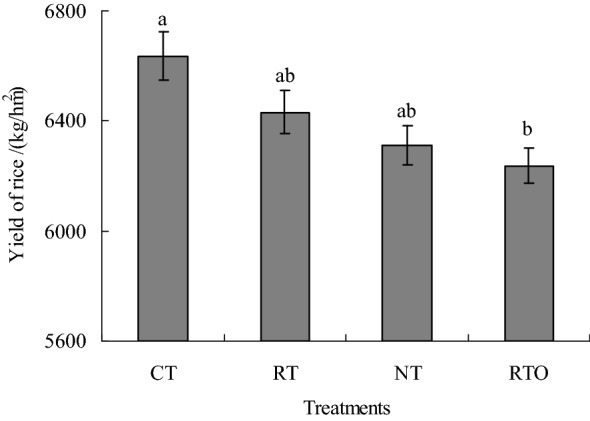
Effects of different tillage treatments on grain yield of late rice. *CT* conventional tillage with crop residue incorporation; *RT* rotary tillage with crop residue incorporation; *NT* no-tillage with crop residue retention; *RTO* rotary tillage with crop residue removed as control. Error bars represent standard error of mean. Different smaller letters were indicated significant difference at *p* < 0.05 level.

There had no significant (*p* > 0.05) difference in grain yield of late rice between NT, RT and RTO treatments (Fig. 2). Compared with RTO treatment, grain yield with CT treatment were significant (*p* < 0.05) increased. The grain yield with NT, RT and CT treatments increased by 1.21%, 3.13% and 6.40% compared to RTO treatment, respectively.

### Composition and content of phospholipid fatty acids

In the present study, twenty-one specific of PLFAs with chain length ranging from C12 to C19 were identified with CT, RT, NT and RTO treatments (Table 2). This results showed that total PLFAs content with CT, RT and NT treatments were significant higher (*p* < 0.05) than that of RTO treatment. The results indicated that PLFAs of aC15:0, C16:0, iC17:0, C19:0c9,10 were dominant in soil microbial community, which were accounted for 15.79–21.27%, 13.66–16.40%, 8.66–11.43% and 8.68–15.56% of total PLFAs content, respectively. The ranged of total PLFAs content with CT, RT, NT and RTO treatments were from 26.47 to 54.76 ng g^−1^. The PLFAs dominant in soil microbial community (aC15:0, C16:0, iC17:0, C19:0c9,10) with CT and RT treatments were significant (*p* < 0.05) increased, compared with RTO treatment. However, the C16:1ω6c in soil microbial community with CT and NT treatments were significant (*p* < 0.05) increased, compared with RT and RTO treatments.

**Table 2:** Effects of different tillage management on soil phospholipid fatty acids content in a double-cropping rice field.

PLFAs	Treatments			
	CT	RT	NT	RTO
C12:0	1.22 ± 0.04a	0.83 ± 0.02b	0.72 ± 0.02c	0.45 ± 0.01d
C14:0	2.13 ± 0.06a	1.35 ± 0.04b	1.14 ± 0.03c	0.63 ± 0.02d
C14:0 2OH	1.45 ± 0.04a	1.15 ± 0.03b	0.76 ± 0.02c	0.54 ± 0.01c
C15:0	2.13 ± 0.06a	1.94 ± 0.06b	1.75 ± 0.05c	1.13 ± 0.03c
aC15:0	8.73 ± 0.25a	7.55 ± 0.22b	6.44 ± 0.18c	5.63 ± 0.16d
iC15:0	1.14 ± 0.03a	0.94 ± 0.03b	0.64 ± 0.02c	0.35 ± 0.01d
C16:0	7.62 ± 0.22a	6.53 ± 0.19b	5.62 ± 0.16c	4.34 ± 0.13d
C16:1ω6c	1.72 ± 0.05a	1.14 ± 0.04b	1.84 ± 0.04a	1.07 ± 0.03b
C16:1ω6t	2.35 ± 0.06a	1.86 ± 0.05b	1.33 ± 0.04c	1.54 ± 0.04d
C17:1ω8c	1.45 ± 0.04a	1.22 ± 0.04b	0.86 ± 0.03c	0.63 ± 0.01d
aC17:0	2.45 ± 0.07a	1.33 ± 0.04b	1.15 ± 0.04c	0.56 ± 0.02d
iC17:0	6.26 ± 0.18a	4.14 ± 0.12b	3.35 ± 0.10c	2.43 ± 0.08d
aC18:0	1.94 ± 0.06a	2.65 ± 0.06b	1.23 ± 0.04c	1.46 ± 0.04d
C18:1ω9t	2.34 ± 0.07c	3.14 ± 0.09b	3.82 ± 0.11a	1.12 ± 0.03d
C18:2ω6	2.84 ± 0.08a	2.13 ± 0.06b	1.85 ± 0.05c	1.26 ± 0.03d
C18:2ω7c	2.25 ± 0.06a	1.64 ± 0.05b	1.15 ± 0.03c	0.54 ± 0.02d
cyc19:0	1.11 ± 0.03a	0.84 ± 0.02b	0.63 ± 0.02c	0.34 ± 0.01d
C19:0c9,10	5.63 ± 0.16a	7.44 ± 0.21b	3.26 ± 0.10c	2.45 ± 0.08d
Total PLFAs (ng/g)	54.76 ± 1.62a	47.82 ± 1.38b	37.54 ± 1.04c	26.47 ± 0.76d

Different lowercase letters in the same line were indicated significantly difference at *p* < 0.05 level.

The same as below.

### Soil microbial community structure

The results indicated that CT treatment had the highest G^+^ bacteria, G^−^ bacteria PLFAs content with 42.35 and 34.64 μg g^−1^, respectively. The NT treatment had the highest value of G^+^ bacteria/G^−^ bacteria PLFAs with 1.35, and the value of G^+^ bacteria/G^−^ bacteria PLFAs with NT treatment were significant (*p* < 0.05) higher than that of CT, RT and RTO treatments. The fungi PLFAs content (23.65 μg g^−1^) and value of fungi/G^+^ bacteria + G^−^ bacteria (0.31) with CT treatment were significant (*p* < 0.05) higher than that of RT, NT and RTO treatments (Table 3).

**Table 3:** PLFAs (μg g^-1^) in Gram-positive bacteria (G^+^ bacteria), Gram-negative bacteria (G^−^ bacteria), fungi and the ratio among them with different tillage treatments.

Items	Treatments			
	CT	RT	NT	RTO
Gram-positive bacteria (G^+^ bacteria)	42.35 ± 1.22a	40.76 ± 1.17ab	38.62 ± 1.11b	29.53 ± 0.99c
Gram-negative bacteria (G^−^ bacteria)	34.64 ± 0.99a	32.75 ± 0.94a	28.58 ± 0.85b	22.34 ± 0.61c
G^+^ bacteria/ G^−^ bacteria	1.22 ± 0.05b	1.24 ± 0.04b	1.35 ± 0.04a	1.32 ± 0.04b
Fungi	23.65 ± 0.68a	19.53 ± 0.56b	16.76 ± 0.48c	8.63 ± 0.24d
Fungi/ G^+^ bacteria + G^−^ bacteria	0.31 ± 0.01a	0.27 ± 0.01b	0.25 ± 0.01b	0.15 ± 0.01c

### Diversity of soil microbial community

The results showed that Richness indices and McIntosh indices with CT treatment were significant higher (*p* < 0.05) than that of RTO treatment, and the order of Richness indices and McIntosh indices with different tillage treatments were showed as following CT > RT > NT > RTO. Compared with RTO treatment, Richness indices and McIntosh indices with CT treatment were increased by 10.94% and 33.68%, respectively. And the Shannon index with CT, RT and NT treatments were significant higher (*p* < 0.05) than that of RTO treatment (Table 4). But there had no significant (*p* > 0.05) difference in Shannon index between CT, RT and NT treatments.

**Table 4:** Effects of different tillage treatments on diversity of soil microbial community in a double-cropping rice field.

Treatments	Items		
	Richness indices	Shannon indices	McIntosh indices
CT	15.82 ± 0.42a	2.55 ± 0.11a	6.35 ± 0.18a
RT	15.47 ± 0.41ab	2.46 ± 0.10a	6.14 ± 0.16ab
NT	15.13 ± 0.40ab	2.38 ± 0.09a	5.78 ± 0.15b
RTO	14.26 ± 0.36b	2.05 ± 0.06b	4.75 ± 0.12c

### Soil microbial phospholipid fatty acids and principal component analysis

**Figure 3:**
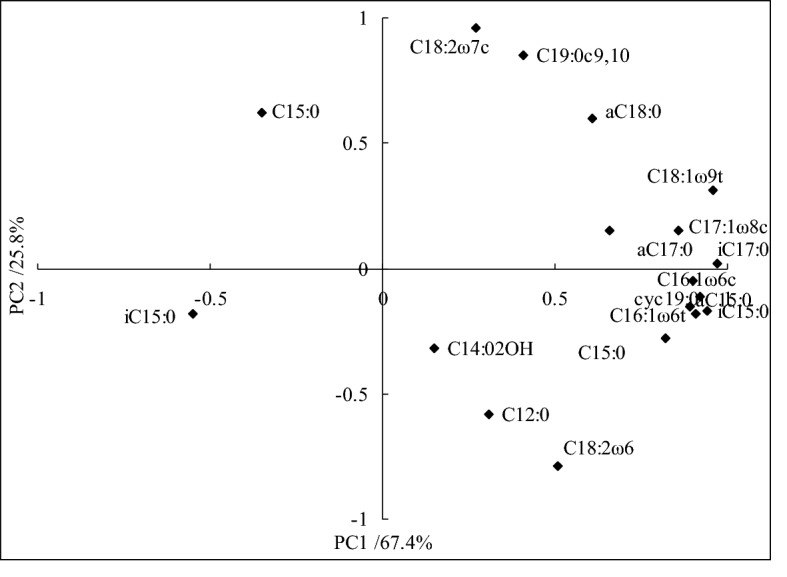
PCA of loading value for individual phospholipids fatty acids with different tillage treatments.

Principal component analysis (PCA) results showed that variability in PLFAs with different tillage treatments were explained by identified fatty acids (Fig. 3). This results indicated that PC1 and PC2 were account for 67.4% and 25.8% of the variation, respectively. In addition to C12:0, C14:0 2OH and C18:2ω6 were included in PC1 and PC2, most of cyclic fatty acids and unsaturated fatty acids were included in PC1 and fatty acids and fungus were included in PC2.

**Figure 4:**
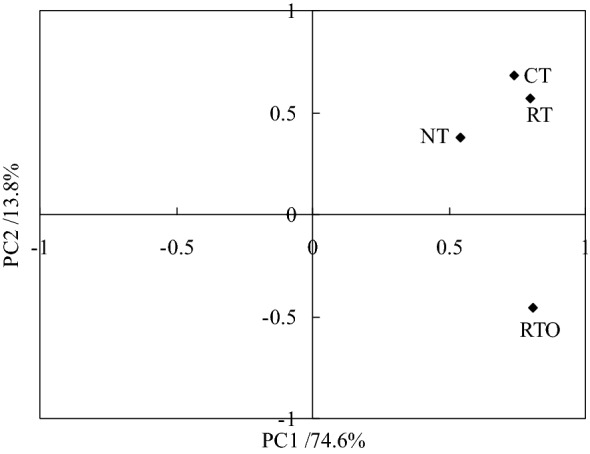
PCA showing variation in PLFAs composition with different tillage treatments. *CT* conventional tillage with crop residue incorporation; *RT* rotary tillage with crop residue incorporation; *NT* no-tillage with crop residue retention; *RTO* rotary tillage with crop residue removed as a control.

The results showed that PC1 and PC2 account for 74.6% and 13.8% of the variation, respectively (Fig. 4). The PLFAs composition with CT, RT, NT and RTO treatments were located in the right-hand side of the plot, PLFAs composition with CT, RT and NT treatments were located in the first quadrant, whereas PLFAs composition with RTO treatment were located in the second quadrant. The results indicated that RT and CT treatments were formed a cluster, with positive scores for PC1 and PC2, whereas the cluster with RTO treatment were away from CT, RT and NT treatments and had negative scores for PC2. Therefore, the results indicated that there had differences in PLFAs composition between CT, RT, NT and RTO treatments.

### Correlation between microbial characteristic and microbial diversity

The results revealed there had significant (*p* < 0.05) positive correlation between value of fungi/G^+^ bacteria + G^−^ bacteria and PLFAs, G^+^ bacteria, G^−^ bacteria, available N and available K contents. And the value of fungi/G^+^ bacteria + G^−^ bacteria had significant (*p* < 0.01) positive correlation with total C, total N contents and grain yield of rice. However, there had significant (*p* < 0.01) negative correlation between value of G^+^ bacteria/G^−^ bacteria and PLFAs, G^+^ bacteria, G^−^ bacteria, fungi and Total K contents. There had significant (*p* < 0.01) positive correlation between Richness indices, Shannon indices and PLFAs, G^+^ bacteria, G^−^ bacteria and fungi contents. There had significant (*p* < 0.05) positive correlation between McIntosh indices and G^+^ bacteria, G^−^ bacteria and fungi contents (Table 5). There had no significant (*p* > 0.05) correlation between Richness, Shannon and McIntosh indices and soil nutrient content, grain yield of rice, whereas the value of fungi/G^+^ bacteria + G^−^ bacteria had significant (*p* < 0.01) positive correlation with soil total C, total N contents and grain yield of rice (Table 5). Therefore, there had significant correlation between soil microbial characteristic and soil microbial diversity.

**Table 5:** Correlation of soil microbial characteristics, microbial diversity indices with soil nutrient and grain yield of rice.

Items	Total PLFAs	G^+^ bacteria	G^−^ bacteria	Fungi	Total C	Total N	Total P	Total K	Available N	Available P	Available K	Grain yield
G^+^ bacteria/G^−^ bacteria	−0.75**	−0.86**	−0.92**	−0.74**	0.33	0.25	0.15	−0.75**	−0.22	−0.15	0.24	−0.34
Fungi/ G^+^ bacteria + G^−^ bacteria	0.51*	0.53*	0.56*	0.84*	0.91**	0.94**	0.21	−0.13	0.57*	−0.16	0.65*	0.86**
Richness indices	0.76**	0.83**	0.78**	0.86**	0.14	0.15	0.52	0.50	0.33	0.41	0.23	0.18
Shannon indices	0.69**	0.76**	0.78**	0.85**	0.12	0.16	0.47	0.42	0.31	0.32	0.25	0.30
McIntosh indices	0.41	0.56*	0.51*	0.58*	0.18	0.13	0.43	0.44	0.37	0.31	0.22	0.16

**, * were indicated significantly difference at *p* < 0.05 and *p* < 0.01 level, respectively.

## Discussion

### Effects of tillage managements on soil microbial community structure

In the previous studies, these results found that soil physicochemical characteristics were varied through change in soil microbial diversity and community under different agricultural managements conditions^10,26^. In the present study, this results showed that soil particulate organic carbon (POC) and dissolved organic carbon (DOC) contents were significantly increased by taken tillage and crop residue incorporation managements, the main reason were attributed to that soil labile organic carbon pool and soil microorganism activity were increased by taken tillage and crop residue incorporation managements, suggested that combined applied with tillage and crop residue incorporation managements had beneficial effects on soil microorganism activity by provided available soil carbon substrate source and suitable soil ecological environment, which were agree with the previous results^27^. Soil microbial plays a vital role in cycling of soil nutrient, which were provided major soil nutrient for crop growth^28^. The PLFAs content in soil microbial community were obvious affected under different soil environment conditions^21^. In the present study, this results showed that soil chemical characteristics with tillage and crop residue incorporation managements (CT, RT and NT) were increased, the reason maybe attributed to that crop residue were incorporated into paddy soil with tillage management, and large number of organic substance were incorporated into soil sublayer of paddy field, which were provided higher soil nutrient and suitable soil ecological environment for soil microbial growth and multiplying, then soil bacterial community structure were enhanced, which were agree with previous results^29^. On the other hand, late rice yield with CT, RT and NT treatments increased by 1.21–6.40% compared to RTO treatment, the main reason maybe attributed to that provided more soil nutrient and suitable soil ecological environment for rice growth under taken tillage and crop residue incorporation conditions, which were consistent with previous results of Yadav et al. (2019)^30^, who suggested that rice yield increased by 5.1–5.3 Mg ha^−1^ by taken tillage and crop residue incorporation practices. In the present study, this results indicated that soil PLFAs content with CT, RT and NT treatments were significantly higher than that of RTO treatment, suggested that applied with crop residue were provided large number of nutrient and substrate for soil microorganism growth and multiplying^15^, which were consistent with previous results of Helgason et al. (2009)^9^, who proved that soil microbial PLFAs content with crop residue treatments increased by 26–58% base on long-term field experiment condition. Furthermore, soil microbial community growth were limited by taken no-tillage and crop residue managements, suggested that soil nutrient contents (DOC and POC contents) and soil ecological environment were changed (Table 1, Fig. 1), and resulted in soil total PLFAs content with NT treatment were significantly lower than that of CT and RT treatments in paddy field^7,9^.

In this study, the PCA results indicated that percentage of cyclopropane fatty acids (cy17:0, cy19:0) with CT and RT treatments were increased, while the percentage of unsaturated fatty acids with CT and RT treatments were decreased, the main reason may be attributed to that crop residue added into paddy soil that soil G^−^ bacteria microbial activities were promoted under combine applied with tillage condition^9^. Furthermore, the lower percentage of unsaturated fatty acids with CT and RT treatments were connected with more soil nutrient content and benefit soil ecological environment^4^. For soil bacteria, the cy17:0, cy18:0 and cy19:0 contents were generally reconsidered as early indicators in change of soil ecological environment, soil bacteria contents were enhanced under disadvantage conditions, including lower soil nutrient content and soil oxygen, soil acidic, and so on^31^. In the present study, this results indicated that cy17:0, cy18:0 and cy19:0 contents with CT, RT and NT treatments were higher than that of RTO treatment, proved that growth of soil bacteria were promoted under crop residue added into paddy soil condition, but soil bacteria growth were restricted under without crop residue input condition for that lower soil nutrient content. However, there were still needed further to investigate the mainly factors that influencing on soil PLFAs content in the double-cropping rice field of southern China.

In the present study, our results showed that soil Richness, Shannon and McIntosh indices with CT and RT treatments were obvious increased. The main reason may be attributed to that soil microorganism were moderate disturbance, soil physical properties, soil nutrient and moisture contents were improved^15,16^, the competitive niche were excluded and selection mechanism were decreased among different soil microorganism population. Therefore, soil microorganism structure diversity were promoted, soil Richness, Shannon and McIntosh indices were also increased^7^. On the other hand, there had similar soil physical properties between conventional tillage and rotary tillage, such as soil texture and soil moisture content. Soil texture and soil moisture content were important factors that influencing on soil pore connectivity, and it were significantly correlated with soil Shannon and Simpson indices^26^, which were in similar with previous results of Yu et al. (2013)^32^. It were benefit strategy for promoting soil bacteria multiplying with less soil disturbances under no-tillage with crop residue retention condition than that of rotary tillage with crop residue removed management, which result in less soil disturbance and provide suitable soil nutrient content and soil ecological environment for soil bacteria growth and multiplying^9,10^. Therefore, our results indicated that soil bacteria community structure and diversity were obvious varied through changed in soil physical properties, soil nutrient and moisture contents by taken crop residue managements.

### Effects of tillage managements on soil microbial characteristics

Soil PLFAs content were generally used as identify individual species of soil fungi and bacteria for that PLFAs were major constituent of cell membranes. Some results indicated that soil bacterial community structure and diversity were measured by using molecular biology technologies, including Illumina MiSeq sequencing, ^13^C-PLFA and PLFA-stable-isotope probing^33^. During different molecular biology technologies, soil bacterial community structure and diversity were usually investigated by using PLFA-base method. Therefore, the analysis method of soil PLFAs content were provided for researcher’s further insight into the structure of bacteria and eukaryotic microorganisms^33^. In the previous studies, these results suggested that soil G^+^ bacteria, G^−^ bacteria and fungi were generally reconsidered as early indicators in change of soil bacteria community structure and diversity by applied with PLFAs method^10^. Therefore, the effects of different tillage and crop residue incorporation practices on soil bacterial community structure and diversity under the double-cropping rice cropping system in southern of China were investigated by using PLFAs method in the present paper. In this study, this results indicated that soil G^+^ bacteria, G^−^ bacteria PLFAs contents with CT and RT treatments were increased, compared to NT and RTO treatments. The reason may be attributed to that it were provide larger amount of soil available substrate for soil microbial growth and multiplying, there had significantly positive influenced on soil G^+^ bacteria and G^−^ bacteria contents by taken tillage and crop residue incorporation managements^9^. The value of soil G^+^ bacteria/G^−^ bacteria PLFAs with NT treatment were highest, which were in similar with the previous results of Wang et al. (2017)^11^, who discovered that value of soil G^+^ bacteria/G^−^ bacteria PLFAs with no-tillage increased by 18.0% compared to conventional tillage, the reason for this phenomenon may be included: (1) soil fungal phospholipid fatty acids (C16:1ω6c and C18:1ω9t) content were tested^34^; (2) it were closely correlated to soil pH^19^. However, the value of soil G^+^ bacteria/G^−^ bacteria PLFAs with CT and RT treatments were decreased, suggested that soil G^−^ bacteria were increased with change of soil environment, such as copiotrophic condition^35^.

Previous studies demonstrated that value of soil fungi/ G^+^ bacteria + G^−^ bacteria were generally reconsidered as early indicators in change of soil microorganisms community and soil ecological environment^6,9^. In the present study, these results indicated that soil fungi PLFAs content and value of fungi/G^+^ bacteria + G^−^ bacteria with CT treatment were higher than that of NT, RT and RTO treatments, which were similar with previous results of Wang et al. (2017)^11^, who proved that soil fungi PLFAs content with conservation tillage practice increased by 61–227% compared to no-tillage practice, which suggested that applied with conventional tillage and crop residue incorporation management were provide larger number of soil available substrate and suitable soil ecological environment for soil microbial growth in the double-cropping rice field, that soil fungal growth were stimulated by taken crop residue with high C/N ratio and then the value of soil fungi/G^+^ bacteria + G^−^ bacteria were increased^11^. Therefore, it was useful strategy for maintaining or improving the soil sustainable production ability in the double-cropping rice field by taken conventional tillage and crop residue incorporation managements.

In the previous studies, these results demonstrated that soil microbial community characteristics were closely correlated to tillage and crop residue managements^10,26^. In this study, there had significantly correlation between soil microbial characteristics and soil microbial diversity. This results also indicated that value of soil fungi/G^+^ bacteria + G^−^ bacteria had significantly positive correlation with soil total N, total C contents and rice yield, which were similar with previous results of Wang et al. (2016)^26^, which found that soil microbial community structure were varied by taken different tillage managements^9,33^. The reason may be attributed to that availability of soil nutrient and substrate, rice growth were increased with crop residue added into paddy field management, which were similar with previous results^36^, who also suggested there had significantly correlation between soil fungi and SOC contents in agricultural field.

## Conclusions

In the present study, the results indicated that soil chemical characteristic and grain yield of rice were increased by short-term combined application of tillage with crop residue incorporation management. Meanwhile, this results showed that total PLFAs, fungal PLFAs contents and value of fungi/G^+^ bacteria + G^−^ bacteria were increased under combined application of tillage with crop residue incorporation condition. The results found that PLFAs of aC15:0, C16:0, iC17:0, C19:0c9,10 were dominant component in soil microbial community with CT, RT, NT and RTO treatments. The proportion of G^+^ bacteria were higher than that of G^−^ bacteria in paddy soil under the same tillage treatment condition. Principal component analyses indicated that content of most PLFAs were increased with CT, RT and NT treatments, which were suggested that the diversity of soil microbial community in paddy field were increased by combined application of tillage with crop residue incorporation management. Therefore, it were found that combined application of conventional tillage and rotary tillage with crop residue incorporation were benefit management for improving soil microbial community in the double-cropping rice field of southern China.
